# Use of Pyriproxyfen to Induce Oogenesis in Diapausing *Megacopta cribraria* (Heteroptera: Plataspidae), and Evaluation of Pyriproxyfen-Induced Eggs for Rearing the Parasitoid *Paratelenomus saccharalis* (Hymenoptera: Scelionidae)

**DOI:** 10.3390/insects13010089

**Published:** 2022-01-13

**Authors:** Cory Penca, Nicholas C. Goltz, Amanda C. Hodges, Norman C. Leppla, Joseph E. Eger, Trevor R. Smith

**Affiliations:** 1 Entomology and Nematology Department, University of Florida, 1881 Natural Area Drive, Steinmetz Hall, Gainesville, FL 32611, USA; achodges@ufl.edu (A.C.H.); ncleppla@ufl.edu (N.C.L.); 2 Department of Plant Science and Landscape Architecture, University of Connecticut, 1380 Storrs Road, Unit 4115, Storrs, CT 06269, USA; nick.goltz@uconn.edu; 3 Florida State Collection of Arthropods, P.O. Box 147100, Gainesville, FL 32614, USA; jeeger811@gmail.com (J.E.E.); trevor.smith@freshfromflorida.com (T.R.S.); 4 Florida Department of Agriculture and Consumer Services, Division of Plant Industry, 1911 SW 34th St., Gainesville, FL 32608, USA

**Keywords:** kudzu bug, diapause, juvenile hormone, biological control, egg parasitoid

## Abstract

**Simple Summary:**

Rearing of many hymenopteran egg parasitoids requires a reliable supply of host eggs. The parasitoid *Paratelenomus saccharalis* can be reared on eggs produced from field collected kudzu bug, *Megacopta cribraria*, however field-collected hosts obtained during reproductive diapause do not readily produce eggs and must be reared under a long day-length photoperiod to terminate diapause. In this study we found that an exogenous application of pyriproxyfen was able to terminate diapause, leading to a significant increase in egg production. The eggs produced by pyriproxyfen-treated *M. cribraria* were accepted by the egg parasitoid *Paratelenomus saccharalis*, however parasitoid emergence was reduced when compared to eggs from untreated hosts. When the effects of pyriproxyfen treatment on egg production and parasitoid emergence were evaluated together, the net increase in parasitoid yield due to pyriproxyfen treatment was approximately 87%. This method has the potential to increase parasitoid yield and reduce production costs in egg parasitoid rearing programs.

**Abstract:**

The mass rearing of hymenopteran egg parasitoids requires an abundant supply of host eggs. The onset of reproductive diapause and subsequent decline in egg production poses a challenge for parasitoid rearing when using host colonies augmented by field-collected insects. We investigated the application of pyriproxyfen, a juvenile hormone analog, to induce oviposition in diapausing adult kudzu bugs, *Megacopta cribraria* (Fabricius) (Heteroptera: Plataspidae), and the use of eggs produced by pyriproxyfen-treated kudzu bugs to rear the egg parasitoid, *Paratelenomus saccharalis* (Dodd) (Hymenoptera: Scelionidae). The effects of pyriproxyfen and photoperiod treatments on host mortality, egg production, and rates of parasitoid eclosion from the eggs were used to calculate the parasitoid yield for the different treatment regimes. A combination of pyriproxyfen and a long-day photoperiod increased the parasitoid yield by 87% compared to acetone and a long-day photoperiod. The general applicability of JH-analog mediated egg production for parasitoid rearing is discussed.

## 1. Introduction

Hymenopteran egg parasitoids are frequently employed in biological control programs against a variety of native and adventive pest species [1,2,3,4,5]. Recently, egg parasitoids have been the subject of investigation for the control of invasive heteropterans, including *Halyomorpha halys* (Stål) (Pentatomidae), *Bagrada hilaris* (Burmeister) (Pentatomidae), and *Megacopta cribraria* (Fabricius) (Plataspidae) [6,7,8,9,10]. A reliable supply of host eggs is required for conducting research on egg parasitoids, while large quantities of host eggs are essential for augmentative releases. The use of field collected insects as a source of host eggs is not feasible on a year-round basis for many Heteroptera due to reproductive diapause, as egg production is terminated during this period [11,12]. The most common means for terminating diapause in a laboratory setting involves exposing field collected specimens to an artificial long-day photoperiod; however, this approach has several drawbacks, including not all species or individuals within a species respond to photoperiod alone and that a significant lag time may occur before the artificial photoperiod can induce oviposition.

Long-day photoperiods serve as the environmental cues to terminate diapause in many Heteroptera, with the physiological response being the resumed production of juvenile hormone (JH) [12,13]. As such, the long-day photoperiod requirement can be bypassed by direct application of JH or JH analogs. The JH analog pyriproxyfen terminates diapause in several species of Heteroptera, resulting in the rapid production of eggs from specimens previously in diapause [14,15,16]. To our knowledge, the use of a JH-analog to produce host eggs, which are then used to successfully rear egg parasitoids, has not been attempted.

The hymenopteran egg parasitoid *Paratelenomus saccharalis* (Dodd) (Scelionidae) is a biological control agent of interest due to its known association with the invasive kudzu bug, *Megacopta cribraria* (Fabricius) [17]. *Megacopta cribraria* was discovered near Atlanta, Georgia in 2009 and has spread rapidly north to Maryland, south to Florida, and west to Arkansas and Louisiana [18,19,20]. *Megacopta cribraria* is a pest of soybean in southeastern U.S. but has a strong preference for kudzu vine, *Pueraria montana* Lour (Merr.) variety *lobata* (Willd.) (Fabaceae), an invasive weed distributed widely in the region [21,22]. Yield loss in soybeans can reach 60% under heavy infestation levels in the absence of control measures [23]. *Paratelenomus saccharalis* was imported into the U.S. for evaluation under quarantine but has since been found in the wild in Florida and other southeastern states where *M. cribraria* is present [6,24]. In Japan the rate of parasitism of *M. cribraria* by *P. saccharalis* has been observed to range from 22% to 45% [17]. In the southeastern United States, rates of parasitism in soybean have been observed to be as high as 58% in organic soybean and approximately 25% in conventional soybean, with parasitism rates increasing during the season partially due to a decline in the overall abundance of eggs [25,26].

The mass rearing of *P. saccharalis* as a biological control agent of *M. cribraria* requires a consistent and abundant supply of host eggs. Field-collected adults are often used to augment colonies of *M. cribraria* because of their abundance and ease of collection; however, it is difficult to rely on field-augmentation with *M. cribraria* when the overwintering adult generation is transitioning into diapause. Diapause in *M. cribraria* is thought to be primarily driven by photoperiod, with the population declining when reared under photoperiods shorter than 12L:12D and egg production ceasing when the daylength was reduced to 8 h or less [27]. As photoperiods below 12 h are present for much of the year reproductive diapause can be a substantial obstacle when field collected specimens are used for host egg production. In this study, we evaluated the application of pyriproxyfen to terminate diapause in *M. cribraria* and increase egg production and determined if pyriproxyfen-induced host eggs produce viable offspring. Finally, we estimated the parasitoid yield resulting from pyriproxyfen treatments using a model that combines the treatment effects on host fecundity and parasitoid eclosion in order to determine whether exogenous application of pyriproxyfen can be utilized to significantly increase parasitoid production.

## 2. Materials and Methods

### 2.1. Megacopta cribraria Collection

Adult *M. cribraria* were collected from kudzu in Alachua County, Florida (29.8055° N, 82.5301° W) on two dates, 17 August 2017 and 26 September 2017. The day length at the time of collection was 13 h 9 min, and 11 h 59 min, during the August and September collections, respectively. Average temperatures over the 14 days preceding collection were 27.03 °C (22.78–33.42 °C) in August and 25.18 °C (19.43–32.50 °C) in September (60 cm. daily average, daily min.–daily max. measurements from the Florida Automated Weather Network Station in Alachua, FL, USA). More than 1000 *M. cribraria* were collected with a sweep net and held in 0.23 m^3^ plexiglass containers provisioned with kudzu vine cuttings in an environmental chamber at 25 °C and 65% RH with a 10L:14D photoperiod for 24 h before treatment. Twenty females from each collection date were dissected to determine their reproductive status. Dissection was conducted by separating dorsal and ventral halves of the abdomen and pulling out the ovaries under a dissection microscope. Water was used to assist in flushing out the ovaries so that they could be examined. All dissected females had regressed ovarioles without mature oocytes, indicating that they were in a state of reproductive diapause [16,28].

### 2.2. Pyriproxyfen and Photoperiod Treatments

At 24 h following field collection, specimens were removed from the plexiglass containers and treated with pyriproxyfen (Chem Service, Westchester, PA, USA) dissolved in >99.5% acetone (Sigma-Aldrich, St. Louis, MO, USA) to a concentration of 0.1% or 1.0% pyriproxyfen or treated with acetone alone. A 2 μL drop was applied to the ventral abdomen of each male and female *M. cribraria* and allowed to evaporate. The treated insects were placed in 1 L clear plastic containers with vented lids (Uline, Pleasant Prairie, WI, USA). Each insect container contained 20 female and 10 male *M. cribraria.* Each container had a piece of filter paper on the bottom, a 10.7 by 20.8 cm paper towel (Kimberly-Clark, Irving, TX, USA) hung on the edge to provide an oviposition substrate, and 4 cuttings (12–16 cm) of pigeon pea, *Cajanus cajan* (L.) (Fabaceae) for food. The cuttings were placed in moist rockwool (Grodan, Roermond, The Netherlands) set inside a shallow plastic dish and changed twice weekly.

Each insect was given one of the three chemical treatments and exposed to either of two different photoperiod regimes, resulting in 6 unique treatment combinations (0.1% pyriproxyfen + 16L:8D, 0.1% pyriproxyfen + 10L:14D, 1.0% pyriproxyfen + 16L:8D, 1.0% pyriproxyfen + 10L:14D, acetone + 16L:8D, acetone + 10L:14D). The photoperiod was controlled in two separate environmental chambers (25 °C 65% RH) set to either 16L:8D or 10L:14D and 12 insect containers (4 per chemical treatment) were placed into each chamber. The resulting experimental design included 4 replicates of 30 *M. cribraria* (20F:10M) for each of the 6 treatment combinations. The experiment was repeated separately for each collection (August and September).

### 2.3. Megacopta cribraria Survival, Oviposition and Egg Viability

Each insect container was checked daily and all eggs and dead insects were removed. Records were kept on the number and sex of dead insects, egg clutches, and eggs per clutch for each experimental replicate. Survival of female *M. cribraria* during the study was visualized via Kaplan–Meier survival curves for qualitative comparisons and estimates of median survival time. The effect of collection month on survival time was analyzed via an accelerated failure time (AFT) model using the Weibull distribution and the R function ‘survreg’ [29]. The effects of chemical treatment (acetone, 0.1% pyriproxyfen and 1.0% pyriproxyfen) and photoperiod (short-day and long-day) were estimated separately for the August and September studies via the same AFT survival analysis. Model estimates for the treatment covariates in the August and September studies were transformed to hazard ratios relative to the short-day + acetone treatment to assist with interpretation.

The influence of chemical treatment and photoperiod on egg production was estimated using a negative binomial generalized linear model with the long-day + acetone treatment set as the referent (intercept) group. The short-day + acetone treatments were not included when modeling the September results as the repeating zero counts produced an error under the negative binomial model due to under-dispersion. This error was corrected by using a hurdle model in which the process for generating the zero counts was modeled via a binomial distribution, with the negative binomial distribution used to model the counts. Estimates of egg production, and contrasts between August and September studies and treatment levels, were conducted using estimated marginal means with Tukey’s adjustment for multiple comparisons [30,31].

To evaluate egg eclosion, clutches less than 24 h old were placed in separate clear 37 cm^3^ plastic vials with a snap top lid (Uline, Pleasant Prairie, WI, USA) and held in a growth chamber set at 25 °C and 65% RH with a 16L:10D photoperiod. Clutches were selected for *M. cribraria* eclosion continuously throughout the 28-day study period based on availability with a minimum of 10 clutches per treatment. The number of 1st instar *M. cribraria* that emerged from each clutch was recorded.

### 2.4. Parasitism of Eggs from Treated Females of Megacopta cribraria

*Paratelenomus saccharalis* were sourced from a colony maintained in a growth chamber (25 °C, 65% RH, 16L:8D), at the Florida Department of Agriculture and Consumer Services, Division of Plant Industry in Gainesville, Florida. The colony was established with parasitoids obtained from *M. cribraria* collected from the same kudzu patch as the *M. cribraria* used in this study and was continually replenished with wild caught parasitoids. To determine whether *P. saccharalis* could be reared on pyriproxyfen-induced eggs, clutches of at least 15 eggs less than 24 h old were obtained for each treatment from the September collection and fixed individually to a 1 cm by 4 cm piece of card-stock paper. An aspirator was used to transfer 2–4 presumably mated *P. saccharalis* females into each vial containing an *M. cribraria* egg card. A 1 cm^2^ piece of honey-soaked tissue paper was added to each vial as a food source for the parasitoids. The vials were placed in a growth chamber maintained at 25 °C, 65% RH, and a 16L:8D photoperiod. Parasitoid-exposed eggs were monitored for at least 28 days and the sex and number of the emerged parasitoids or emerged *M. cribraria* were recorded. Eggs with a darkened appearance may have been parasitized (Gardner et al., 2013), and were examined under a dissection microscope for the presence of partially developed or non-emerged parasitoids. Non-emerged parasitoids were used to determine if parasitism had occurred and were not included in the analysis of parasitoid viability. A total of 69 egg clutches were exposed to the parasitoids, with the number of replicates per treatment varying based on the resulting fecundity of the treated females. As the acetone + 10L:14D treatment did not result in eggs, no parasitoid exposures were conducted for this treatment.

Eclosion of *M. cribraria* from treated and control eggs was modeled via logistic regression with a binary outcome (viable/non-viable) assigned to each egg, with a weight term assigned based on clutch size. Viability of *P. saccharalis*, recorded as percent emergence from exposed eggs, was modeled using the same approach as used for viability of *M. cribraria*, with percent viability recorded for the eggs in each clutch, and a weight term assigned based on clutch size. Contrasts of model estimates of eclosion rates between treatments were made using estimated marginal means with Tukey’s adjustment for multiple comparisons.

### 2.5. Parasitoid Yield Estimates

The effect of treatment on parasitoid yield was estimated using a Monte-Carlo simulation. For each run of the simulation, a value for the number of eggs per replicate and probability of parasitoid emergence was drawn from the probability distribution produced by the modeled treatment response obtained previously. The product of these two values is equivalent to the number of parasitoids produced from that simulation (i.e., parasitoid yield). Simulations were repeated 2000 times for each treatment. The yield estimates for the simulations of each treatment were compared via a generalized linear model to determine treatment effects on parasitoid yield. Post-hoc tests to evaluate differences in parasitoid yield between treatments were conducted using estimated marginal means with Tukey’s adjustment for multiple comparisons.

## 3. Results

### 3.1. Survival of Adult Megacopta cribraria

**Figure 1:**
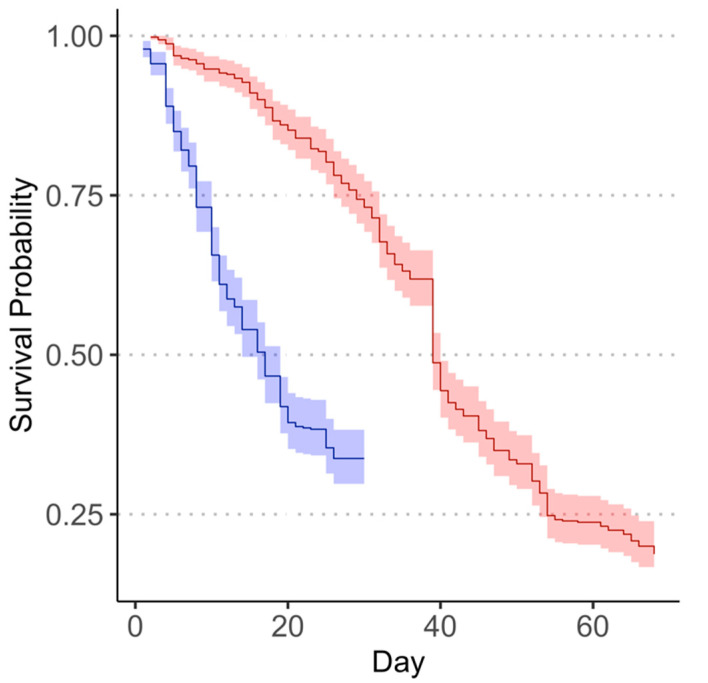
Survival curve showing differences in the instantaneous survival probability between the August (blue) and September (red) collections. Shaded areas represent 95% confidence intervals. Day zero represents the application of chemical treatments.

Survival of the *M. cribraria* females differed significantly between the August and September collections (Weibull distributed AFT model: *X*^2^ = 139.34, *df* = 1, *p* < 0.0001). *Megacopta cribraria* female survival declined more rapidly in the August collection (median time to death: 17 days; 95% C.I.: 14–19 days) than in the September collection (median time to death: 39 days; 95% C.I.: 39–40 days) (Figure 1). The probability of mortality for female *M. cribraria* collected in August was approximately 2.72 times that of the September collection as indicated by the converted hazard ratio for the August collection (95% CI: 2.32–3.21).

**Figure 2:**
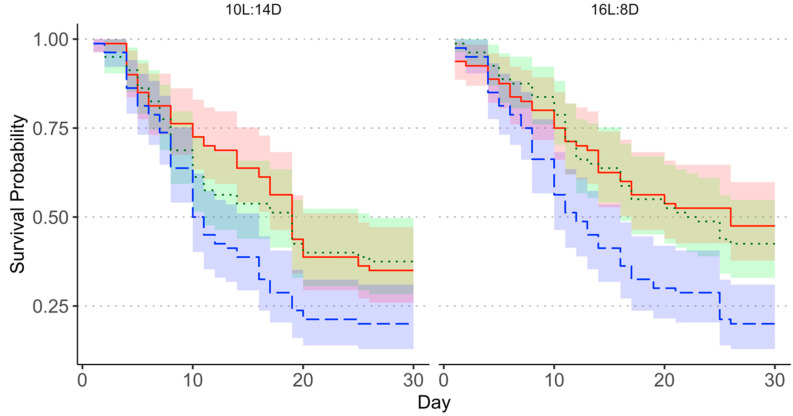
Survival curve for the August collection showing differences in the instantaneous survival probability between the acetone (red solid line), 1.0% pyriproxyfen (blue dashed line), and 0.1% pyriproxyfen (green dotted line) treatments, separated by photoperiod treatment. Shaded areas represent 95% confidence intervals.

When the August collection was analyzed separately, Kaplan–Meier survival curves for female *M. cribraria* failed to significantly diverge between treatments, likely because population-wide background mortality occurred before the mortality effects associated with the treatments (Figure 2). The Weibull distributed AFT model fit was significant (*X*^2^ = 29.98, *df* = 3, *p* < 0.0001). The 1.0% pyriproxyfen treatment had a significant negative effect on survival; however, the coefficients for the 0.1% treatment and long-day treatment were not significant predictors of mortality in the August collection (Table 1). The probability of mortality was 1.9 times higher for the 1.0% pyriproxyfen treatments than the short-day + acetone treatment (Hazard ratio: 1.90, 95% CI: 1.45–2.49). Hazard ratios for the 0.1% pyriproxyfen treatment and long-day photoperiod did not differ significantly from 1.

**Figure 3:**
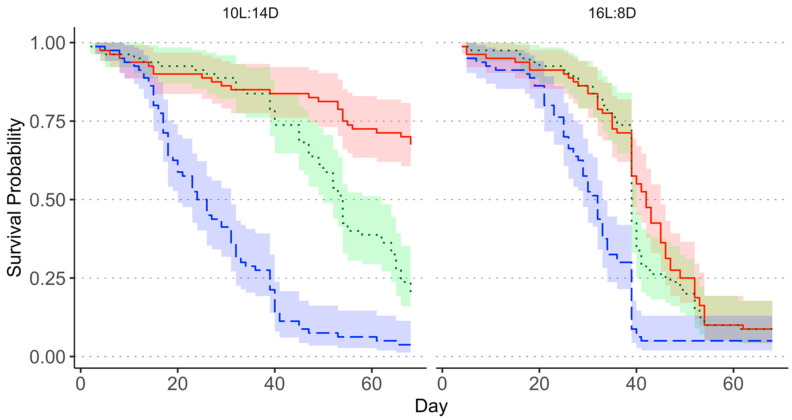
Survival curve for the September collection showing differences in the instantaneous survival probability between the acetone (red solid line), 1.0% pyriproxyfen (blue dashed line), and 0.1% pyriproxyfen (green dotted line), separated by photoperiod treatment. Shaded areas represent 95% confidence intervals.

Differences in *M. cribraria* mortality between treatments were apparent in the September collection, with survival curves diverging after approximately 20 days (Figure 3). The Weibull distributed AFT model fit was significant (*X*^2^ = 162.6, *df* = 3, *p* < 0.0001). All treatment covariates were significant and had a negative effect on survival, with the 1.0% pyriproxyfen treatment having the strongest effect (Table 1). The hazard ratio for the 1.0% pyriproxyfen treatment was 4.06 (95% CI: 3.12–5.28), indicating that this treatment increased the probability of mortality by a factor of 4.06 relative to the short-day + acetone treatment. The hazard ratios for the 0.1% acetone treatment (1.67, 95% CI: 1.29–2.16) and the long-day photoperiod (1.85, 95% CI: 1.51–2.28) were also demonstrative of an increase in mortality and were of comparable strength.

### 3.2. Egg Production and Eclosion of Megacopta cribraria and Paratelenomus saccharalis

Egg production differed significantly between females of *M. cribraria* collected in August (462 eggs produced) and September (7101 eggs produced) (*df* = 1,38, *F* = 55.84, *p* < 0.001). When egg production was standardized based on cumulative days of female survival, the relative difference between the August (3554 female days lived) and September females (15,780 female days lived) was reduced (0.13 vs. 0.45 eggs per female days lived for the August and September studies, respectively). The lengthened photoperiod and pyriproxyfen applications at 0.1% and 1.0% induced oogenesis and oviposition in females from both the August and September collections, whereas the short-day + acetone treated insects from both months did not produce eggs.

**Figure 4:**
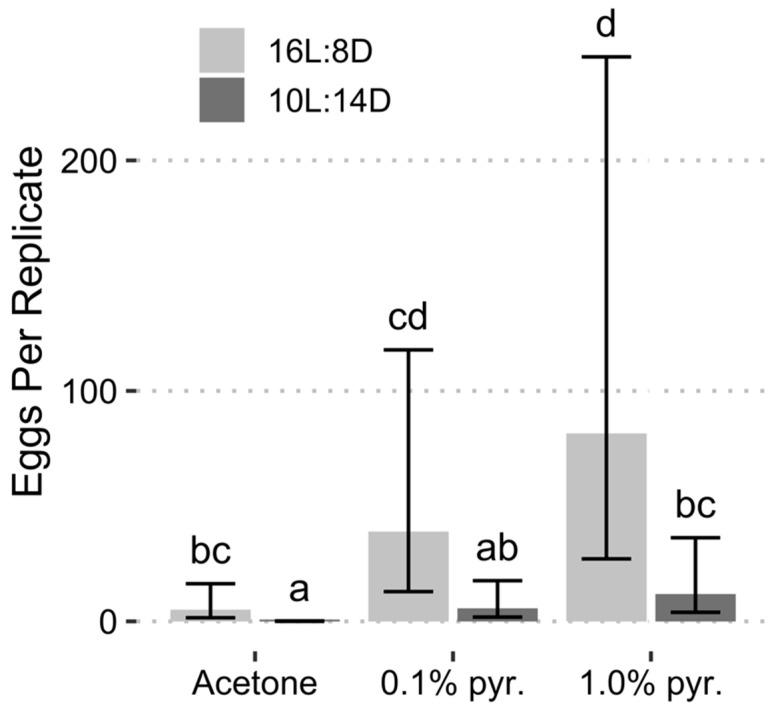
Egg production separated by chemical treatment (pyr. = pyriproxyfen) and photoperiod from the August collection. Error bars represent 95% confidence intervals. Bars with the same letter are not significantly different (α = 0.05, estimated marginal means with Tukey’s method of adjustment for multiple comparisons).

In the August collection, both photoperiod (*F* = 9.294, *df* = 1.20, *p* = 0.0063) and chemical treatment (*F* = 5.737, *df* = 2.20, *p* = 0.0107) were significant predictors of egg production. All pyriproxyfen treatments resulted in significant increases in egg production, whereas a short-day photoperiod was associated with a reduction in egg production (Table 2). When the photoperiod was held constant 16L:8D or 10L:14D, the differences between egg production in the 0.1% and 1.0% pyriproxyfen treatments were not significantly different from each other and only the 1.0% pyriproxyfen treatments were significantly different from the acetone treatments at their respective photoperiods (Figure 4). Variance between replicates was high, likely due to the confounding effect of increased mortality in the August collection.

**Figure 5:**
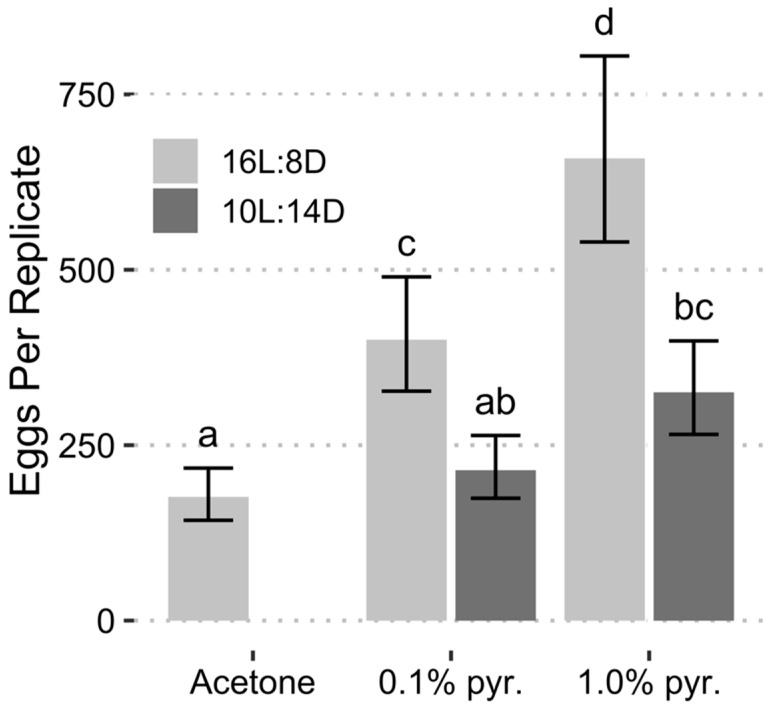
Egg production separated by chemical treatment (pyr. = pyriproxyfen) and photoperiod from the September collection. Error bars represent 95% confidence intervals. Bars with the same letter are not significantly different (α = 0.05, estimated marginal means with Tukey’s method of adjustment for multiple comparisons).

In the September collection both photoperiod (*F* = 25.18, *df* = 1.16, *p* = 0.0001) and chemical treatment (*F* = 13.83, *df* = 2.16, *p* = 0.0003) were significant predictors of egg production. All coefficients from the regression model were significant (Table 2). The model intercept represents the long-day + acetone treatment, which averaged 176 eggs for the 20 female: 10 male replicates. The addition of 1.0% pyriproxyfen increased egg production by a factor of 3.70 relative to the long-day + acetone treatment, while an increase of 2.32 was attributed to the 0.1% pyriproxyfen treatment. When the females were reared under the short-day photoperiod, egg production was reduced by approximately 51% relative to the long-day photoperiod. Differences in estimated egg production between treatment combinations in the September collection followed a trend similar to that observed in the August collection, however, within-treatment variance was reduced, and contrasts were more pronounced (Figure 5). Only the short-day + 0.1% pyriproxyfen treatment failed to produce significantly more eggs than that of the long-day + acetone control.

Eggs produced by pyriproxyfen treated *M. cribraria* had significantly lower *M. cribraria* eclosion rates (<2.5%) when compared to eggs produced by the long-day-acetone treatment (28.1%) (Table 3). *Paratelenomus saccharalis* were observed ovipositing in *M. cribraria* eggs shortly after exposure. Emergence of *P. saccharalis* from exposed eggs was highest for the eggs produced under long-day conditions and treated with acetone or 0.1% pyriproxyfen, with 34.7% and 28.8% parasitoid emergence, respectively (Table 3). A shorter photoperiod and higher dose of pyriproxyfen both resulted in significantly decreased parasitoid viability.

### 3.3. Parasitoid Yield

**Figure 6:**
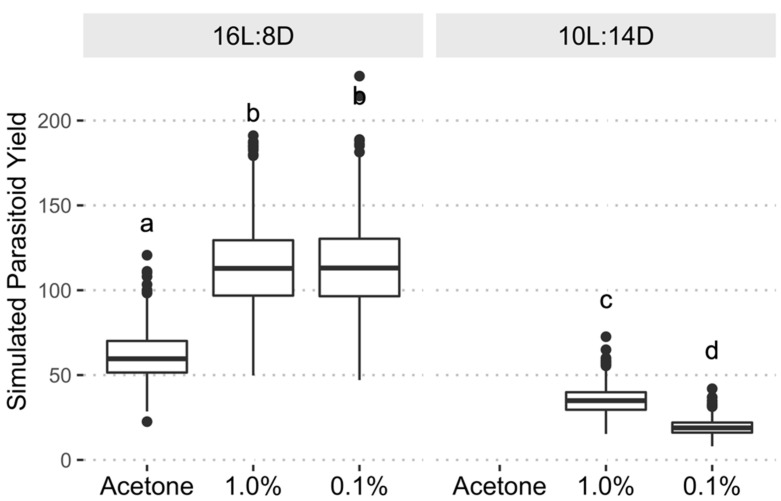
Estimated parasitoid yield from bootstrap simulations (*n* = 2000) using model estimates of egg production and parasitoid viability. The long-day photoperiod significantly increased parasitoid yield over short-day treatments, while within the long-day photoperiod the pyriproxyfen treatments produced similar parasitoid yields and yielded significantly more parasitoids than the acetone treatment. Boxplots with the same letter are not significantly different (α = 0.05, estimated marginal means with Tukey’s method of adjustment for multiple comparisons).

The combined treatment effects on host fecundity and parasitoid eclosion resulted in the long-day + acetone treatment producing an average of 61.06 (95% CI: 38.01–90.20) parasitoids per 20 host females in the simulation; whereas the long-day + 0.1% pyriproxyfen and long-day + 1.0% pyriproxyfen produced an average of 114.28 (95% CI: 73.57–164.04) and 113.74 (95% CI: 70.50–169.58) parasitoids per 20 host females, respectively. The resulting gain in the long-day + pyriproxyfen treatments is an 86% to 87% increase in average parasitoid production when compared to the long-day acetone treatment. Differences in parasitoid yield between the 0.1% and 1.0% pyriproxyfen treatments were not significantly different under long-day rearing conditions, while under short-day rearing conditions the 1.0% pyriproxyfen produced significantly more eggs than the 0.1% pyriproxyfen treatment (Figure 6). Under short-day rearing conditions the addition of pyriproxyfen at either dose was not sufficient to increase parasitoid yield beyond that of the long-day + acetone control. The results of the simulated parasitoid yields, which combined the estimates for egg production and parasitoid viability, suggest that the addition of exogenous pyriproxyfen at either dose can provide a significant increase in parasitoid production when rearing is conducted under a long-day photoperiod.

## 4. Discussion

This study demonstrated that treatment of *M. cribraria* with pyriproxyfen, in combination with a long-day photoperiod, can increase the availability of host eggs suitable for mass rearing the egg parasitoid *P. saccharalis*. A long-day photoperiod, coupled with 0.1% pyriproxyfen resulted in an 87% increase in parasitoid yield compared to manipulation of photoperiod alone, whereas 1% pyriproxyfen resulted in a slightly lower increase in parasitoid yield. In addition, while pyriproxyfen treatment induced oviposition in *M. cribraria,* these eggs had significantly lower rates of eclosion when compared to *M. cribraria* eggs from non-pyriproxyfen treated *M. cribraria*. The net benefit of pyriproxyfen treatment was reduced when not used in combination with a long-day photoperiod, and our results suggest that, while pyriproxyfen application can induce oviposition, it does not completely replicate the effects achieved from photoperiod manipulation. Because mass production of egg parasitoids requires simultaneous mass production of host eggs, the ability to increase egg production per host can significantly improve the efficiency of the parasitoid rearing program.

Our study showed differences in survival time between specimens collected in August and September. We suspect this may be a result of different generations of insects being present in each collection. Specifically, the August collection may have included adults from the non-overwintering generation whereas the September collection may have been primarily comprised of overwintering adults. Megacopta cribraria is thought to have two generations per year in the USA, and the period of overlap between these generations is expected to occur during the autumn months, which aligns with our collection period [22]. Temperature differences between collection dates were minimal, and insects were reared at 25 °C, which has been shown to be an optimal temperature for egg production [32]. Importantly, because the differences in longevity were seen across all treatments it is likely that the cause for the difference in survival time was not related to treatments themselves. Efforts were made to ensure the collection process was similar between collection months, with the 24 h acclimation period intended to reduce the influence of stress resulting from the collection process. However, it is possible that the collection, transport and handling of field collected specimens may have contributed to some of the mortality observed in the study.

This research provides an example of an alternative use for a JH analog, terminating host diapause to increase host egg production and parasitoid yield. While juvenile hormone analogs have primarily been used to control pests through disruption of the insect life cycle, they also have been useful in manipulating insect behavior. Application of the JH analog methoprene, for example, has increased silk production in *Bombyx mori* by at least 24% [33,34]. In the commercial production of baculovirus, the exogenous application of a JH analog resulted in a 2.7 to 2.9-fold increase in baculovirus yield, largely due to the supernumerary molt of the lepidopteran host [35]. In studies with *H. halys*, pyriproxyfen was used to induce egg production, resulting in eggs with extremely low viability [16]. When these pyriproxyfen-induced eggs were presented to the parasitoid, *Trissolcus japonicus* (Ashmead), they were readily accepted for oviposition, but no viable parasitoids were produced. The present study showed that, in the case of *P. saccharalis,* eggs produced by pyriproxyfen-treated *M. cribraria* are suitable for parasitoid development, although a decline in parasitoid viability was observed.

In addition to host and parasitoid rearing, altering the photoperiod and applying JH have potential applications for management of pests in the field. Artificial manipulation of photoperiod as a means of diapause interference has been demonstrated as a potential control method for the European corn borer *Ostrinia nubilalis* (Hübner) and codling moth *Cydia pomonella* (L.) [36]. However, the use of JH analogs to interfere with diapause may be easier to implement than manipulating photoperiods in field scenarios. The diapause-terminating effect of pyriproxyfen could produce a significant reduction in overwintering survival when a population in diapause is exposed to a sufficient dose. Our findings indicated that interruption of diapause by JH negatively affects survival of the treated adult *M. cribraria*. Another possibility is “prepping” a field for the release of an egg parasitoid or predator by applying pyriproxyfen to induce mass oviposition. Augmentative releases in the area, if timed to correspond with the treatment effect, could benefit the released parasitoids by increasing the likelihood that they will encounter suitable host eggs.

The results presented in this study should not be generalized to other host-parasitoid systems without certain conditions being met. First, preliminary, species-specific work must be done to optimize the dose and treatment schedule of the host, and to determine if the parasitoid is able to successfully develop and emerge from the pyriproxyfen-induced eggs. Second, the method described herein is most useful when specimens are readily field collected and have already undergone reproductive diapause. Exogenous IGR applications may increase egg production in non-diapausing specimens, though the benefits should be weighed against any effects on host mortality and egg quality and the described technique may not be worthwhile for host colonies that are already producing eggs. Our study relied on a single application of pyriproxyfen made at the beginning of the observation period, however, multiple applications may be warranted, as this may produce sustained activation of the JH receptor. While pyriproxyfen is noted as having a strong affinity for the JH binding site, the metabolization of pyriproxyfen over the course of the study could have reduced the strength of the signal promoting vitellogenesis and the quality of the resulting eggs [37].

This study demonstrates that the use of pyriproxyfen in combination with an artificial long-day photoperiod can significantly increase egg production. For example, in the September experiment the four replicates of the 1% pyriproxyfen treatment produced a total of 2636 eggs under long-day conditions, whereas the acetone control produced 705 eggs under long-day conditions, and zero eggs under a short-day photoperiod. This large (over 370%) increase in egg productivity was attenuated by the lower rate of parasitoid eclosion in the pyriproxyfen treated eggs, resulting in an 87% increase in parasitoid production. Increased parasitoid yield will influence the required size of the host colony; a more efficient rearing program will require less space and resources, lowering the costs and thus making biological control via egg parasitoids a more viable solution to managing invasive species. Future research focused on increasing the rate of successful parasitoid eclosion from pyriproxyfen-induced eggs would further enhance the yield gains observed in this study, providing a substantial benefit to mass rearing programs for hymenopteran egg parasitoids.

## Figures and Tables

**Table 1:** Regression coefficients and predictors from the AFT survival model. Survival of *Megacopta cribraria* decreased significantly due to 1.0% pyriproxyfen and long-day treatments in the August experiment and by all treatments in the September experiment. The model intercept represents the long-day + acetone treatment, which serves as the referent group.

Collection	Factor	Coefficient	SE	Z	*p*
August	Intercept	3.375	0.099	33.92	<0.0001
	1.0% pyr.	−0.546	0.117	−4.68	<0.0001
	0.1% pyr.	−0.045	0.123	−0.37	0.7130
	Long-day	0.154	0.096	−1.61	0.1072
	Log(scale)	−0.1620	0.049	−3.33	<0.0001
September	Intercept	4.307	0.050	86.07	<0.0001
	1.0% pyr.	−0.621	0.059	−10.48	<0.0001
	0.1% pyr.	−0.226	0.059	−3.85	0.0001
	Long-day	−0.273	0.046	−5.92	<0.0001
	Log(scale)	−0.815	0.042	−19.35	<0.0001

**Table 2:** Regression coefficients representing the influence of pyriproxyfen (pyr.) and photoperiod treatments on egg production. Egg production by *Megacopta cribraria* was significantly greater due to 1.0% pyriproxyfen and long-day treatment compared with the 0.1% and short-day treatment in the August experiment and was increased by all treatments in the September experiment. The model intercept represents the long-day + acetone treatment, which serves as the referent group. Coefficients and errors present on the log scale.

Collection	Factor	Coefficient	SE	Z	*p*
August	Intercept	1.619	0.599	2.70	0.0069
	1.0% pyr.	2.782	0.738	3.77	0.0002
	0.1% pyr.	2.045	0.741	2.76	0.0058
	Short-day	−1.921	0.588	−3.27	0.0011
September	Intercept	5.172	0.107	48.20	<0.0001
	1.0% pyr.	1.299	0.140	9.31	<0.0001
	0.1% pyr.	0.841	0.140	6.01	<0.0001
	Short-day	−0.666	0.104	−6.396	<0.0001

**Table 3:** Percentage of *Megacopta cribraria* eclosion and *P. saccharalis* emergence by treatment group from the September collection.

Treatment		*Megacopta cribraria* ^1^		*Paratelenomus saccharalis*	
Chemical	Photoperiod	Eggs	% Eclosion ± SE	Eggs	% Emergence ± SE
Acetone	10L:14D	-	-	-	-
Acetone	16L:8D	400	28.06 ± 1.91 a	128	34.69 ± 3.34 a
0.1% pyr.	10L:14D	183	0.54 ± 0.54 b	366	8.95 ± 1.42 c
0.1% pyr.	16L:8D	665	2.21 ± 0.56 b	321	28.82 ± 2.13 a
1.0% pyr.	10L:14D	177	0.00 ± 0.00 b	384	10.90 ± 1.50 c
1.0% pyr.	16L:8D	1065	1.48 ± 0.37 b	414	17.36 ± 1.69 b

^1^ 80 females per chemical treatment and photoperiod. Estimates within the same column which share a common letter are not significantly different (α = 0.05, estimated marginal means with Tukey’s method of adjustment for multiple comparisons).

## Data Availability

Data is available upon request from the corresponding author.

## References

[1] Corrêa-Ferreira B.S., Moscardi F. (1996). Biological control of soybean stink bugs by inoculative releases of *Trissolcus basalis*. Entomol. Exp. Appl..

[2] Smith S.M. (1996). Biological control with *Trichogramma*: Advances, successes, and potential of their use. Annu. Rev. Entomol..

[3] Waage J.K., Hassell M.P. (1982). Parasitoids as biological control agents–a fundamental approach. Parasitology.

[4] Mills N. (2009). Egg parasitoids in biological control and integrated pest management. Egg Parasitoids in Agroecosystems with Emphasis on Trichogramma.

[5] Mills N. (2005). Selecting effective parasitoids for biological control introductions: Codling moth as a case study. Biol. Control.

[6] Gardner W.A., Blount J.L., Golec J.R., Jones W.A., Hu X.P., Talamas E.J., Evans R.M., Dong X., Ray C.H., Buntin G.D. (2013). Discovery of *Paratelenomus saccharalis* (Dodd) (Hymenoptera: Platygastridae), an egg parasitoid of *Megacopta cribraria* F. (Hemiptera: Plataspidae) in its expanded North American range. J. Entomol. Sci..

[7] Ruberson J.R., Takasu K., David Buntin G., Eger J.E., Gardner W.A., Greene J.K., Jenkins T.M., Jones W.A., Olson D.M., Roberts P.M. (2013). From Asian curiosity to eruptive American pest: *Megacopta cribraria* (Hemiptera: Plataspidae) and prospects for its biological control. Appl. Entomol. Zool..

[8] Yang Z.Q., Yao Y.X., Qiu L.F., Li Z.X. (2009). A new species of *Trissolcus* (Hymenoptera: Scelionidae) parasitizing eggs of *Halyomorpha halys* (Heteroptera: Pentatomidae) in China with comments on its biology. Ann. Entomol. Soc. Am..

[9] Sforza R.F.H., Bon M.-C., Martel G., Augé M., Roche M., Mahmood R., Smith L., Mason P.G., Gillespie D.R., Vincent C. (2017). Initial evaluation of two native egg parasitoids for the control of *Bagrada hilaris*, and invasive stink bug in Western USA. Proceedings of the 5th International Symposium on Biological Control of Arthropods.

[10] Leskey T.C., Hamilton G.C., Nielsen A.L., Polk D.F., Rodriguez-Saona C., Christopher Bergh J., Ames Herbert D., Kuhar T.P., Pfeiffer D., Dively G.P. (2012). Pest status of the brown marmorated stink bug, *Halyomorpha halys* in the USA. Outlooks Pest Manag..

[11] Davey K.G. (1997). Hormonal controls on reproduction in female heteroptera. Arch. Insect Biochem. Physiol..

[12] Saulich A.K., Musolin D.L. (2012). Diapause in the seasonal cycle of stink bugs (Heteroptera, Pentatomidae) from the Temperate Zone. Entomol. Rev..

[13] Musolin D.L. (2007). Insects in a warmer world: Ecological, physiological and life-history responses of true bugs (Heteroptera) to climate change. Glob. Chang. Biol..

[14] Numata H., Hidaka T. (1984). Termination of adult diapause by a juvenile hormone analogue in the bean bug, *Riptortus clavatus*. Zool. Sci..

[15] Amiri A., Bandani A.R., Darvishzadeh A. (2012). Effects of the insect growth regulators methoxyfenozide and pyriproxyfen on adult diapause in sunn pest *Eurygaster integriceps* (Hemiptera: Scutelleridae). J. Agric. Sci. Tech..

[16] Penca C., Hodges A.C. (2017). Pyriproxyfen treatment terminates *Halyomorpha halys* reproductive diapause, with an indirect mortality effect on its egg parasitoid Trissolcus japonicus. J. Pest. Sci..

[17] Hoshino K., Adati T., Olson D.M., Takasu K. (2017). Seasonal occurrence and interspecific interactions of egg parasitoids of megacopta cribraria (heteroptera: Plataspidae) in Japan. Environ. Entomol..

[18] Suiter D.R., Eger J.E., Gardner W.A., Kemerait R.C., All J.N., Roberts P.M., Greene J.K., Ames L.M., Buntin G.D., Jenkins T.M. (2010). Discovery and distribution of *Megacopta cribraria* (Hemiptera: Heteroptera: Plataspidae) in northeast Georgia. J. Integr. Pest Manag..

[19] Liang W., Tran L., Washington-Allen R., Wiggins G., Stewart S., Vogt J., Grant J. (2018). Predicting the potential invasion of kudzu bug, *Megacopta cribraria* (Heteroptera: Plataspidae), in North and South America and determining its climatic preference. Biol. Invasions.

[20] Eger J.E., Ames L.M., Suiter D.R., Jenkins T.M., Rider D.A., Halbert S.E. (2010). Occurrence of the Old World bug *Megacopta cribraria* (Fabricius) (Heteroptera: Plataspidae) in Georgia: A serious home invader and potential legume pest. Insecta Mundi.

[21] Blount J.L., Buntin G.D., Sparks A.N. (2015). Host preference of *Megacopta cribraria* (Hemiptera: Plataspidae) on selected edible beans and soybean. J. Econ. Entomol..

[22] Zhang Y., Hanula J.L., Horn S. (2012). The biology and preliminary host range of *Megacopta cribraria* (Heteroptera: Plataspidae) and its impact on kudzu growth. Environ. Entomol..

[23] Seiter N.J., Greene J.K., Reay-Jones F.P.F. (2013). Reduction of soybean yield components by *Megacopta cribraria* (Hemiptera: Plataspidae). J. Econ. Entomol..

[24] Medal J., Cruz A.S., Williams K., Fraser S., Wolaver D., Smith T., Davis B.J. (2015). First Record of *Paratelenomus saccharalis* (Hymenoptera: Platygastridae) on Kudzu Bug *Megacopta cribraria* (Heteroptera: Plataspidae) in Florida. Fla. Entomol..

[25] Tillman G., Gaskin J., Endale D., Johnson C., Schomberg H. (2016). Parasitism of *Megacopta cribraria* (Hemiptera: Plataspidae) by Paratelenomus saccharalis (Hymenoptera: Platygastridae) in Organic Soybean Plots in Georgia, USA. Fla. Entomol..

[26] Knight I.A., Roberts P.M., Gardner W.A., Oliver K.M., Reay-Jones F.P.F., Reisig D.D., Toews M.D. (2017). Spatial distribution of *Megacopta cribraria* (Hemiptera: Plataspidae) adults, eggs and parasitism by *Paratelenomus saccharalis* (Hymenoptera: Platygastridae) in soybean. Environ. Entomol..

[27] Xu Z., Cui J., Bi R., Shi S., Gao Y. (2019). Effect of photoperiod on development and reproduction of *Megacopta cribraria* (Hemiptera: Plataspidae). Oil Crop. Sci..

[28] Hodek I. (1971). Termination of adult diapause in *Pyrrhocoris apterus* (Heteroptera: Pyrrhocoridae) in the field. Entomol. Exp. Appl..

[29] Therneau T. (2015). A Package for Survival Analysis in R. R Package Version 3.2-13. https://CRAN.R-project.org/package=survival.

[30] Searle S.R., Speed F.M., Milliken G.A. (1980). Population marginal means in the linear model: An alternative to least squares means. Am. Stat..

[31] Lenth R.V. (2020). Emmeans: Estimated Marginal Means, Aka Least-Squares Means. R Package Version 1.5.3. https://CRAN.R-project.org/package=survival.

[32] Shi S.S., Cui J., Zang L.S. (2014). Development, survival, and reproduction of *Megacopta cribraria* (Heteroptera: Plataspidae) at different constant temperatures. J. Econ. Entomol..

[33] Miranda J.E., de Bortoli S.A., Takahashi R. (2002). Development and silk production by silkworm larvae after topical application of methoprene. Sci. Agric..

[34] Murakoshi S., Chang C.-F., Tamura S. (1972). Increase in silk production by the silkworm, *Bombyx mori* L., due to oral administration of a juvenile hormone analog. Agric. Biol. Chem..

[35] Lasa R., Caballero P., Williams T. (2007). Juvenile hormone analogs greatly increase the production of a nucleopolyhedrovirus. Biol. Control.

[36] Hayes D.K., Sullivan W.N., Oliver M.Z., Schechter M.S. (1970). Photoperiod manipulation of insect diapause: A method of pest control?. Science.

[37] Charles J.-P., Iwema T., Epa V.C., Takaki K., Rynes J., Jindra M. (2011). Ligand-binding properties of a juvenile hormone receptor, methoprene-tolerant. Proc. Natl. Acad. Sci. USA.

